# *Capsicum annuum* transcription factor WRKYa positively regulates defense response upon TMV infection and is a substrate of CaMK1 and CaMK2

**DOI:** 10.1038/srep07981

**Published:** 2015-01-23

**Authors:** Sung Un Huh, Gil-Je Lee, Ji Hoon Jung, Yunsik Kim, Young Jin Kim, Kyung-Hee Paek

**Affiliations:** 1 College of Life Sciences and Biotechnology, Korea University, 1, 5-ga, Anam-dong, Sungbuk-gu, Seoul 136-701, Republic of Korea

## Abstract

Plants are constantly exposed to pathogens and environmental stresses. To minimize damage caused by these potentially harmful factors, plants respond by massive transcriptional reprogramming of various stress-related genes via major transcription factor families. One of the transcription factor families, WRKY, plays an important role in diverse stress response of plants and is often useful to generate genetically engineered crop plants. In this study, we carried out functional characterization of *CaWRKYa* encoding group I WRKY member, which is induced during hypersensitive response (HR) in hot pepper (*Capsicum annuum*) upon *Tobacco mosaic virus* (TMV) infection. CaWRKYa was involved in *L*-mediated resistance via transcriptional reprogramming of *pathogenesis*-*related* (*PR*) gene expression and affected HR upon TMV-P_0_ infection. CaWRKYa acts as a positive regulator of this defense system and could bind to the *W*-box of diverse *PR* genes promoters. Furthermore, we found *Capsicum annuum* mitogen-activated protein kinase 1 (CaMK1) and 2 (CaMK2) interacted with CaWRKYa and phosphorylated the SP clusters but not the MAPK docking (D)-domain of CaWRKYa. Thus, these results demonstrated that CaWRKYa was regulated by CaMK1 and CaMK2 at the posttranslational level in hot pepper.

The plant defense response against pathogen infection is triggered by the recognition of pathogen-associated molecular patterns (PAMPs) or effectors^1^,^2^. When a plant recognizes the PAMP, PAMP-triggered immunity (PTI) activates basal defense such as production of reactive oxygen species (ROS), phytoalexins, and *pathogenesis-related* (*PR*) gene expression to prevent pathogen penetration and proliferation^3^. In contrast, when plant resistance (R) proteins recognize effectors, effector-triggered immunity (ETI) activates stronger and faster resistance responses often accompanied by hypersensitive response (HR) cell death^1^. In both basal defense and *R* gene-mediated resistance, plant mitogen-activated protein kinase (MAPK) cascades play a central role in signal transduction via three-kinase phosphorelay systems composed of the MAPK, MAPK kinase (MKK) and MAPK kinase kinase (MKKK)^4^. Approximately 23 MAPKs, 10 MKKs, and 80 MKKKs exist in *Arabidopsis* and the cascade combinations of the components make complex signal transduction networking^5^. These cascade events affect various cellular processes such as resistance to biotic or abiotic agents and plant development via phosphorylation or protein-protein interaction with diverse target proteins. However, few target proteins of MAPKs have been identified and regulation of downstream signaling pathway is poorly understood.

WRKY transcription factors have a conserved WRKY domain of about 60 amino acids containing a 'WRKYGQK' heptapeptide and zinc finger-like motif that can bind to *W*-box (TTGAC[C/T]) in target genes to regulate gene expression upon abiotic/biotic stress conditions^6^,^7^. In plants, the WRKY family has been identified in almost all the plant species including *Arabidopsis* (74 members), soybean (197 members), poplar (104 members), sorghum (68 members), barley (more than 45 members), and rice (more than 100 members). WRKY proteins are mainly classified into group I, IIa + IIb, IIc, IId + IIe, and III based on sequence homology^8^,^9^,^10^,^11^. The *WRKY* genes from several plant species are highly upregulated by various stimuli such as pathogen infection, wounding stress, and exogenous defense-related plant hormone treatments. WRKYs also participate in defense responses as a positive or negative regulator through regulation of downstream gene expression^7^,^12^. Furthermore, WRKY proteins are activated or inactivated by MAPK-dependent phosphorylation or the conformational change of binding partner protein. Substrates of MAPKs were enriched in WRKY transcription factors and validated phosphorylation of WRKYs by MAPKs^13^,^14^. Recently, it was shown that salicylic acid (SA)-induced protein kinase (SIPK), wound-induced protein kinase (WIPK), and tobacco mitogen-activated protein kinase 4 (NTF4) directly phosphorylated *Nicotiana benthamiana* WRKY8 (NbWRKY8), which is involved in plant innate immunity^14^,^15^. Interestingly, the interaction of NbWRKY8 with MAPKs is dependent on a MAPK-docking domain (D-domain) and phosphorylation efficiency is affected by serine-proline (SP) clusters located in the NbWRKY8 N-terminal region^15^. In *Arabidopsis*, MPK3 and MPK6 can phosphorylate AtWRKY33 upon *Botrytis cinerea* infection and phosphorylated AtWRKY33 activates *phytoalexin deficient 3* (*PAD3*) expression^16^. The NbWRKY8 and AtWRKY33 are classified as group I WRKY family members. Group I members contain conserved D-domain and at least five SP clusters at N-terminal region^17^. Thus, WRKY transcription factors of group I could have a possibility of being substrates of MAPKs.

In barley, *MLA10* encodes a nucleotide binding (NB) and leucine-rich repeat (LRR) type R protein that recognizes a fungal avirulence A10 (AVR_A10_) effector protein and then localizes to the nucleus. The Coiled-coil (CC) domain of MLA10 interacts with the transcriptional repressors HvWRKY1 or HvWRKY2, and then another unknown WRKY protein might activate defense gene expression^18^. The *Arabidopsis* genome encodes the Resistant to *Ralstonia solanacearum* 1-R (RRS1-R) protein which has an N-terminal Toll and interleukin-1 receptor, resistance protein (TIR) NB-LRR domain and C-terminal WRKY DNA binding domain^19^. The *sensitive to low humidity 1* (*slh1*) mutant, which possesses an additional amino acid in the WRKY domain of RRS1-R, exhibits impaired DNA binding affinity and causes activation of defense response and hypersensitive cell death^20^. Thus, WRKY domains might be involved in both basal defense and effector-triggered immunity by R protein.

Hot pepper (*Capsicum annuum* L.) is important crop. The hot pepper plant contains *L* gene alleles which encode CC-NB-LRR type resistance proteins and the *L* gene confers resistance to *Tobacco mosaic virus* (TMV) by restricting virus spread at the primary infection site^21^. Previously, we verified *WRKY* and *MAPK* genes were responding to TMV-P_0_ infection via DNA microarray analysis of a hot pepper expressed sequence tag (EST) database^22^. Specifically, some *WRKY* genes were involved in HR upon TMV-P_0_ infection and positively regulated expression of *PR* genes^22^,^23^,^24^. In this study, we performed functional study of *CaWRKYa* via virus-induced gene silencing (VIGS) and demonstrated CaWRKYa positively regulated *PR* gene expressions upon TMV-P_0_ infection. Furthermore, CaMK1 and CaMK2 directly interacted with and phosphorylated CaWRKYa which contains D-domain and SP clusters at N-terminus. These results indicated that phosphorylation of CaWRKYa by two TMV-P_0_-responsive MAPKs (CaMK1 and CaMK2) could play a role in TMV defense response in hot pepper plant.

## Results

### Sequence analysis of CaWRKYa

**Figure 1:**
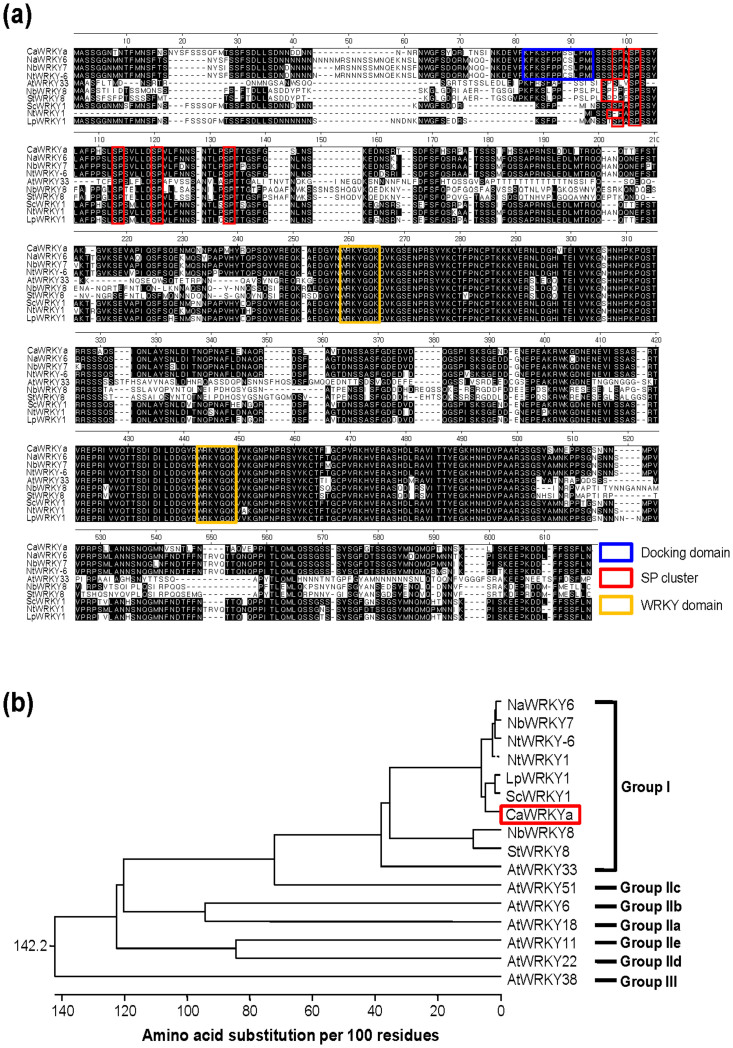
Analysis of CaWRKYa amino acid sequence. (a) CaWRKYa amino acid sequence was aligned with other group I WRKY members from various plants by MegAlign software. Members of group I typically contain two WRKY domains (yellow box). The docking domains (D-domain) for MAPKs are conserved at the N-terminal region of WRKYs (blue box). Some WRKYs do not contain D-domain. At the N-terminal region WRKYs contain some SP clusters which are potential phosphorylation sites by MAPKs (red box). Accession numbers; NaWRKY6 (AAS13440), NbWRKY7 (BAI63295), NtWRKY-6 (BAB61053), NtWRKY1 (BAA82107), ScWRKY1 (AAQ72790), LpWRKY1 (ABI95141), CaWRKYa (AAR26657), NbWRKY8 (BAI63296), StWRKY8 (BAI63294), AtWRKY33 (NP_181381). (b) Phylogenetic analysis of CaWRKYa and other group WRKY members by Clustal W method. Accession numbers; WRKY51 (AED97953), AtWRKY6 (AEE33948), AtWRKY18 (AEE85961), AtWRKY11 (AEE85927), AtWRKY22 (AEE81999), AtWRKY38 (AED93044).

We previously isolated a CaWRKYa cDNA clone from library screening and deduced the amino acid sequence of CaWRKYa, which contains two 'WRKY' domains and thus CaWRKYa was assigned to group I compared with other groups (Fig. 1a). Recently some group I member WRKYs were found to contain MAPK-docking sites (D-domain) and Ser or Thr followed by Pro (SP) clusters at the N-terminus^15^,^17^. By amino acid analysis, we found CaWRKYa contained a D-domain and five SP clusters at N-terminal region (Fig. 1a). CaWRKYa exhibited 91% and 90% amino acid sequence homology with *Solanum chacoense* (Sc) WRKY1 and *Lycopersicon peruvianum* (Lp) WRKY1, respectively (Fig. 1b). LpWRKY1 was phosphorylated by unknown 44 kD and 67 kD protein kinases which are shown to be transiently activated in response to an elicitor-preparation derived from the wilt inducing fungus *Fusarium oxysporum* *lycopersici* (E-FOL)^25^. These results suggest that CaWRKYa is a member of group I WRKY and has putative phosphorylation sites.

### *CaWRKYa* is involved in *L*-mediated resistance upon TMV-P_0_ infection

**Figure 2:**
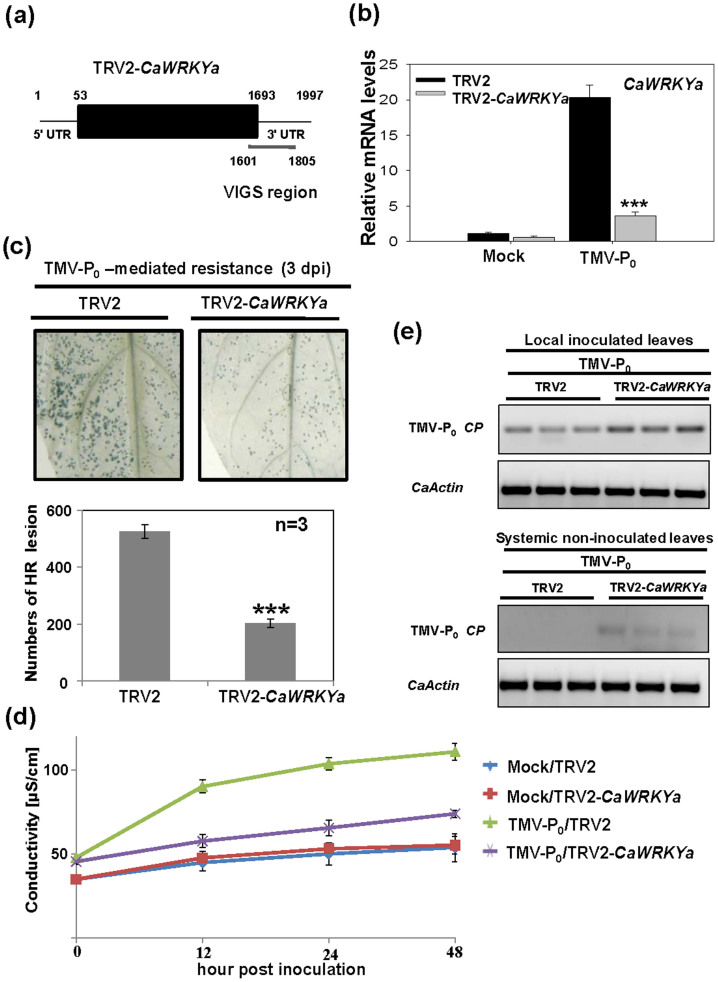
CaWRKYa is involved in *L*-mediated resistance upon TMV-P_0_ infection. (a) A schematic illustration of VIGS region of *CaWRKYa*. Partial 3' UTR fragment of *CaWRKYa* cDNA was used for VIGS. (b) qRT-PCR analysis of *CaWRKYa-*silenced plants. To confirm silencing of *CaWRKYa*, TRV2-*CaWRKYa* and TRV2 control plants were inoculated with TMV-P_0_. After 3 day post inoculation (dpi), total RNA was extracted from three independent samples and qRT-PCR was performed (Student's *t*-tests; ***P < 0.0001). (c) Trypan blue staining analysis for detecting the hypersensitive response (HR) cell death. Numbers of HR were quantified by counting (Student's *t*-tests; ***P < 0.0001). (d) Ion conductivity was measured by electrolyte leakage assay upon TMV-P_0_ inoculation or mock-inoculation in the TRV2 control and TRV2-*CaWRKYa* plants at 3 dpi. The error bars show the mean of the standard deviation (SD) of the replicate samples. (e) RT-PCR analysis of TMV-P_0_-*coat protein* (*CP*) expression in the local inoculated tissue and systemic non-inoculated upper tissue. Total RNA was extracted from three independent samples and RT-PCR was performed.

The roles of *CaWRKYa* in the *L* gene-mediated resistance against TMV-P_0_ infection in hot pepper plants were studied by using VIGS system based on *Tobacco rattle virus* (TRV) vector using a partial fragment of *CaWRKYa* containing 3' untranslated region (UTR) (Fig. 2a). The expression level of *CaWRKYa* was reduced about 67% in TMV-P_0_-infected TRV2-*CaWRKYa* plants compared with the TMV-P_0_-infected TRV2 control plants (Fig. 2b). To investigate whether silencing of *CaWRKYa* affects HR cell death upon TMV-P_0_ infection trypan blue staining and conductivity measurement were performed. At 3 days post-inoculation (dpi), the HR numbers of *CaWRKYa*-silenced plants were reduced by about 40% and ion conductivities were decreased by about 30% compared with TMV-P_0_-inoculated TRV2 plants (Fig. 2c and d). To test whether silencing of *CaWRKYa* affects *L*-mediated resistance upon TMV-P_0_ infection, we performed RT-PCR analysis of TMV-P_0_-*coat protein* (*CP*) gene expression in the local inoculated leaves and systemic non-inoculated upper leaves. In the local inoculated leaves of TRV2-*CaWRKYa* plants, TMV-P_0_-*CP* mRNA was still detected compared with TRV2 control plants (Fig. 2d). In the systemic non-inoculated upper leaves, TMV-P_0_-*CP* was detected only in the *CaWRKYa*-silenced plants but not in the TRV2 control plants (Fig. 2e). Thus, *L*-mediated resistance was affected by silencing of *CaWRKYa*. These results imply that suppression of *CaWRKYa* expression leads to reduced resistance to TMV-P_0_ and CaWRKYa is a component of the *L*-mediated TMV resistance response in hot pepper.

### CaWRKYa positively regulated expression of *PR* genes and some plant hormone related genes upon TMV-P_0_ infection

**Figure 3:**
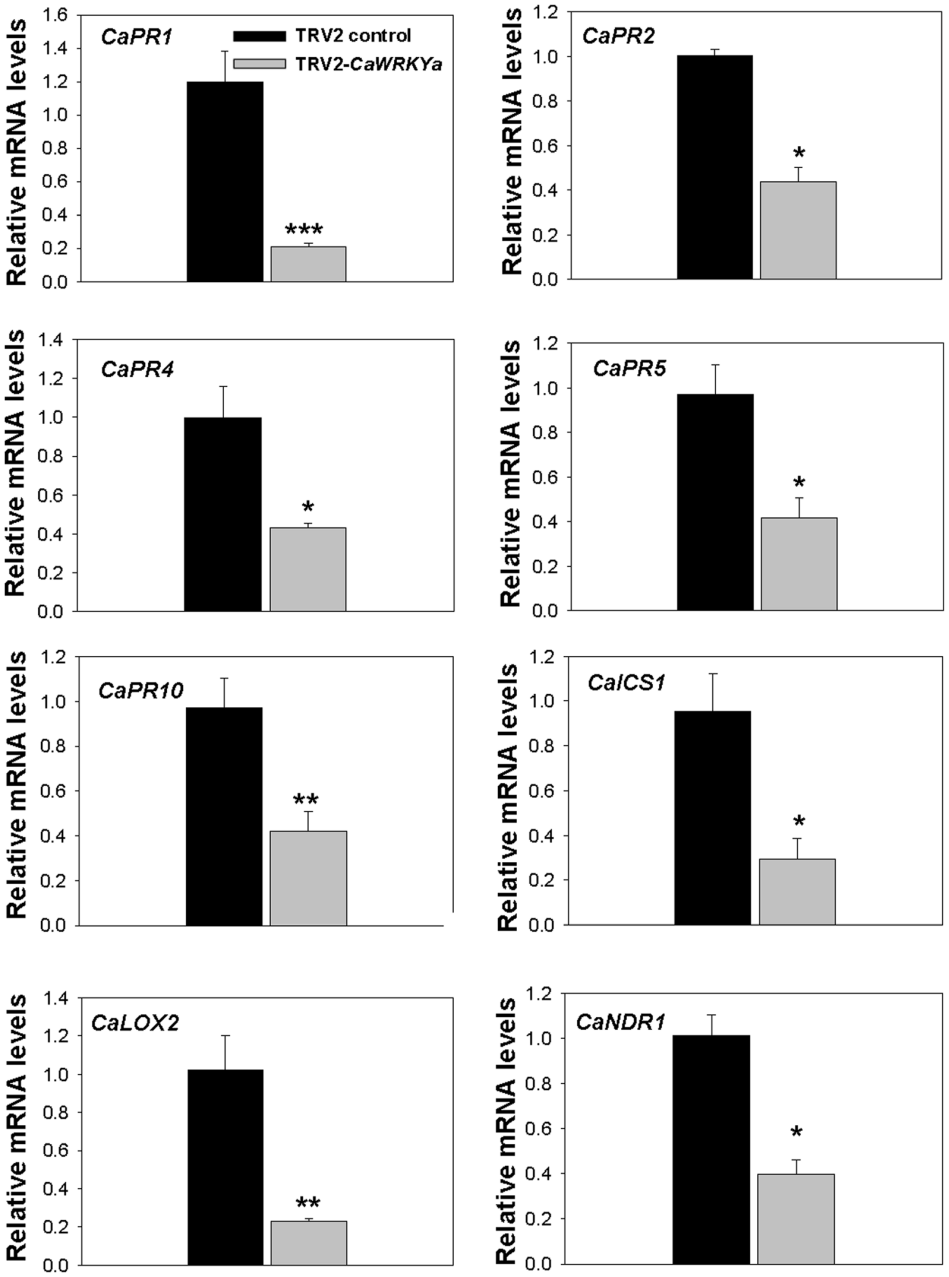
*CaWRKYa* silencing affects some *PR* and defense-related genes expression upon TMV-P_0_ infection. In TMV-P_0_-inoculoated *CaWRKYa*-silenced and TRV2 control plants, qRT-PCR was performed with some *PR* genes and defense-related genes primers. Asterisks indicate significant differences in TMV-P_0_-inoculated TRV2-*CaWRKYa* compared with TRV2 control plants. (Student's *t*-tests; *P < 0.05, **P < 0.001, ***P < 0.0001).

WRKY transcription factors play a crucial role in regulating multiple defense response genes such as *PR* genes^9^. We have shown the *L*-mediated resistance was diminished in hot pepper plants upon TMV-P_0_ infection when the expression of *CaWRKYa* was suppressed in *CaWRKYa*-silenced plants by VIGS (Fig. 2d and e). Furthermore, we showed that CaWRKYa bound to the *W*-box (TTGAC[C/T]) but not to a mutated version *in vitro* (Supplementary Fig. S1a and b). Following infection with TMV-P_0_, we tested whether expression of *PR* genes was affected by suppression of *CaWRKYa* expression. Expressions of *CaPR1*, *2*, *4*, *5* and *10* were strongly reduced in the *CaWRKYa*-silenced plants compared to the TRV2 control plants (Fig. 3). To determine whether CaWRKYa activates these *PR* genes, we checked activity of the *CaPR10promoter*-*GUS* reporter when co-expressing *CaWRKYa-GFP* in tobacco leaves by *Agrobacterium*-mediated infiltration^25^. The activity of *CaPR10pro-GUS* was increased about 37% when it was co-transformed with *CaWRKYa-GFP* compared to GFP (Supplementary Fig. S1c). These results suggest that *CaPR10* might be one of the target genes of CaWRKYa and CaWRKYa might be a positive regulator of other *PR* genes by binding to the *W*-box-containing promoter.

Furthermore, gene expression level of *CaICS1* was also diminished in *CaWRKYa*-silenced plants upon TMV-P_0_ infection (Fig. 3). In general, *PR1*, *PR2*, and *PR5* are involved in salicylic acid (SA)-dependent signaling pathway and *isochorismate mutase 1* (*ICS1*) encodes one of the major enzymes of SA biogenesis^26^. *Non-race-specific disease resistance 1* (*NDR1*), a major regulator of *R* gene-mediated resistance, was also downregulated in the *CaWRKYa*-silenced plant^27^ (Fig. 3). *Lipoxygenase* (*LOX*) genes are involved in jasmonic acid (JA) biogenesis pathway and associated with plant defense^28^,^29^. In *CaWRKYa*-silenced plant, *CaLOX2* induction was reduced (Fig. 3). These data demonstrate that CaWRKYa regulates the expression of defense-related genes and signaling pathway genes. These regulations could contribute to the decreased resistance of *CaWRKYa*-silenced plant to TMV-P_0 _infection.

### *CaMK1* and *CaMK2* are induced during HR to TMV-P_0_ infection

Previously, using hot pepper microarray analysis, we characterized *CaMK1* and *CaMK2* as TMV-P_0_-induced genes during HR in hot pepper^22^. *CaMK1* and *CaMK2* were identified as encoding stress-inducible protein kinases which respond to wounding, UV-C, and cold^30^. However, the function of CaMK1 and CaMK2 in plant immunity was not verified. CaMK1 is classified as group A according to the amino acid sequence analysis (Supplementary Fig. S2)^31^. The amino acid sequence analysis between CaMK1 and LeMPK3 or WIPK showed 95% and 94% identity, respectively (Supplementary Fig. S2). Thus, we speculated that CaMK1 and CaMK2 might have similar function in *L*-mediated plant defense response of hot pepper to TMV infection.

**Figure 4:**
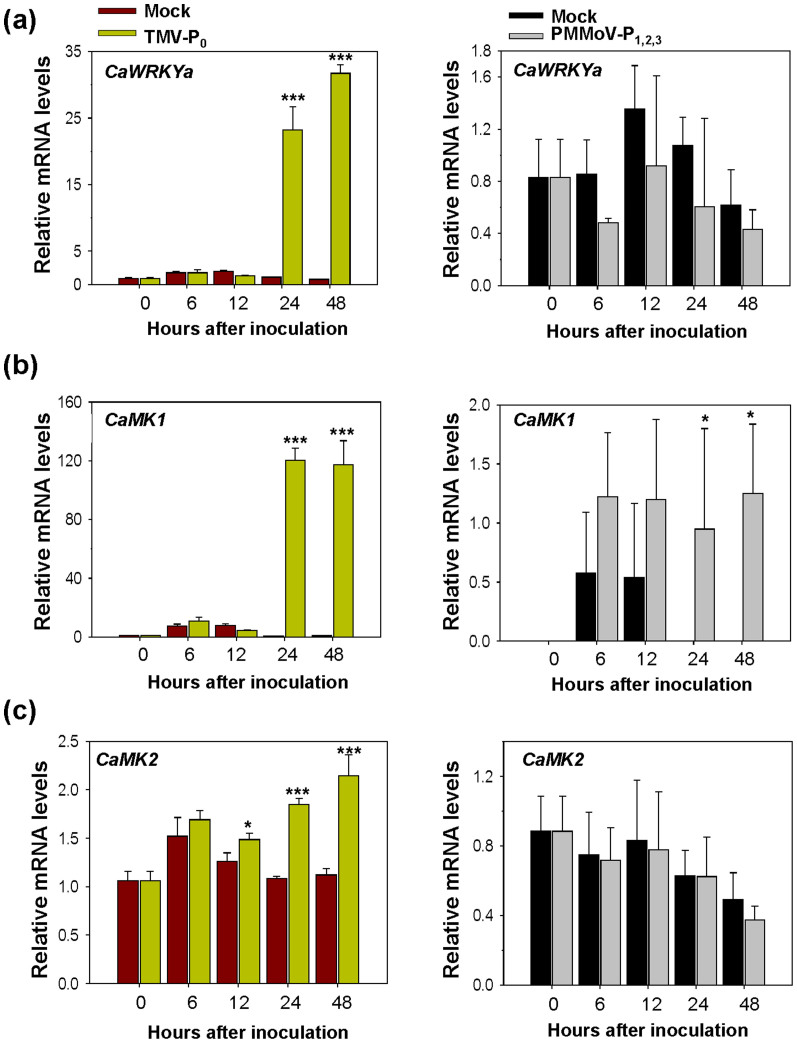
Gene expression analysis of *CaWRKYa*, *CaMK1*, and *CaMK2* upon TMV-P_0_ infection. (a–c) The expanded leaves of six-week-old hot pepper plants were rubbed with TMV-P_0_ or PMMoV-P_1,2,3_. Inoculated samples were harvested at the indicated time points and total RNA was extracted. qRT-PCR was performed with gene-specific primers. These experiments were performed with three independent samples. *CaActin* and *Ca18S* were used as the internal controls. (Student's *t*-tests; *P < 0.05, ***P < 0.0001).

To elucidate CaMK1 and CaMK2 function in *L*-mediated resistance, we checked whether *CaMK1* and *CaMK2* could be induced specifically during HR upon TMV-P_0_ infection using qRT-PCR. We first confirmed that *CaWRKYa* expression level was induced by an avirulent strain, TMV-P_0_, but not by a virulent strain, PMMoV-P_1,2,3_ (Fig. 4a)^32^. The mRNA levels of *CaMK1* and *CaMK2* were increased within 24 h after TMV-P_0_ inoculation (Fig. 4b and c). Thus, *CaMK1* and *CaMK2* are responsive genes to TMV-P_0_ infection. In particular, *CaMK1* gene expression level was highly increased by TMV-P_0_ inoculation at 24 h and slightly induced upon PMMoV-P_1,2,3_ infection (Fig. 4b). Furthermore, mRNA levels of *CaWRKYa* and *CaMK1* were increased at early time points by exogenous application of salicylic acid (SA), methyl jasmonic acid (MeJA), and ethephon (ET) treatments but *CaMK2* mRNA level was not increased (Supplementary Fig. S3a, c, and d). *CaPR1* was used as a positive control for treatments (Supplementary Fig. S3b)^23^. As *CaMK1* expression is induced upon treatment with plant hormones and TMV infection, and *CaMK2* expression is only induced upon TMV infection, this suggests that they play different signaling roles in the plant. Furthermore, we showed that *CaMK1* or *CaMK2* silencing of hot pepper plants exhibited reduced HR lesions upon TMV-P_0_ infection (Supplementary Fig. S4). We thus postulated that function of CaWRKYa might be linked to the CaMK1 and CaMK2 and regulated by CaMK1/CaMK2 via post-translational modification.

### CaMK1 and CaMK2 can interact with CaWRKYa *in vivo*

**Figure 5:**
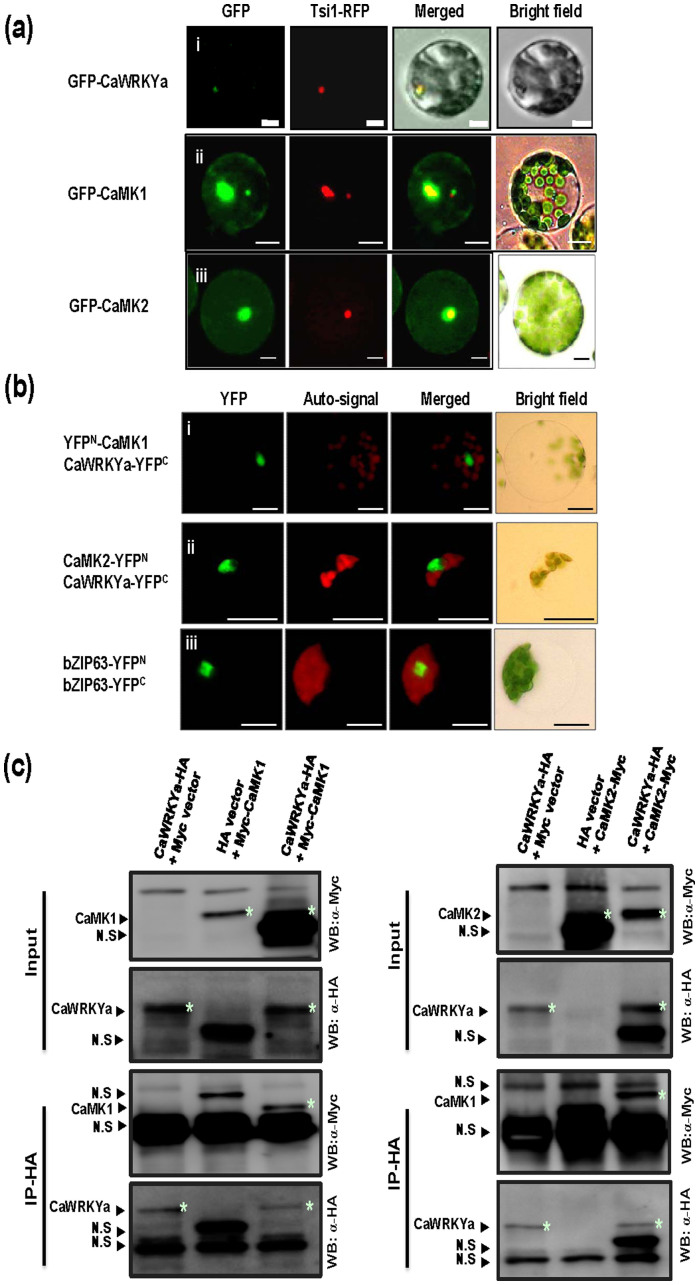
CaWRKYa associates with CaMK1 and CaMK2 *in vivo*. (a) Localization analysis of CaWRKYa, CaMK1, and CaMK2 using GFP in the hot pepper protoplasts. (Panel, i–iii) The combination of *GFP-CaWRKYa*/*Tsi1-RFP*, *GFP-CaMK1*/*Tsi1-RFP*, and *GFP-CaMK2*/*Tsi1-RFP* constructs were cotransformed into the hot pepper protoplasts by PEG-mediated transformation. *Tsi1-RFP* was used as a nucleus localization control. GFP and RFP signals were detected using Axioplan 2 imaging fluorescence microscope. Bars indicate 20 μm. (b) CaWRKYa associates with CaMK1 and CaMK2 in the split YFP analysis. Panel i, Combination of *YFP^N^-CaMK1* and *CaWRKYa-YFP^C^* constructs was introduced into hot pepper protoplasts by PEG-mediated transformation. Bars indicate 20 μm. Panel ii, Combination of *CaMK2-YFP^N^* and *CaWRKYa-YFP^C^* constructs was introduced into hot pepper protoplasts by PEG-mediated transformation. Reconstructed YFP fragments exhibited YFP signals in the nucleus. Red signals indicate chloroplast auto-signals. Panel iii, Combination of *bZIP-YFP^N^* and *bZIP-YFP^C^* constructs was introduced into hot pepper protoplasts by PEG-mediated transformation. bZIP transcription factor forming homodimer complex in the nucleus was used as a positive control for BiFC (c) CaWRKYa can associate with CaMK1 and CaMK2 in the co-IP. Co-IP was performed using anti-HA beads and then detected by anti-Myc and -HA antibodies. Combinations of *Myc-CaMK1* and *CaWRKYa-HA* or *CaMK2-Myc* and *CaWRKYa-HA* were introduced into the hot pepper protoplasts by PEG-mediated transformation. Combinations of *CaWRKYa-HA* and *Myc* empty vector or *Myc-CaMK1*/*CaMK2-Myc* and *HA* empty vector were used as a negative control. Specific signal bands are indicated by asterisks. N.S indicates non-specific bands.

CaWRKYa can be classified as a WRKY group I member, having two WRKY domains, and D-domain and SP clusters at the N-terminal region (Fig. 1a). Many substrates of MAPK were identified as transcription factors, such as WRKYs, in the nucleus to regulate gene expression^14^,^33^,^34^. To determine whether CaWRKYa is localized in the nucleus, as other WRKY proteins are, a construct expressing *GFP*-*CaWRKYa* fusion was transformed into hot pepper protoplasts along with *Tsi1-RFP* construct as a nucleus-target control^35^,^37^. Signal of GFP-CaWRKYa predominantly was merged with Tsi1-RFP signal in the nucleus although the CaWRKYa did not contain any obvious nuclear localization signal (NLS) (Fig. 5a, panel i). Thus, CaWRKYa localizes to the nucleus in hot pepper protoplasts. Most of interacting proteins generally co-localize to the same or interactive subcellular space. Consequently, we tested the subcellular localization of CaMK1 and CaMK2. The construct of *GFP* fused to N-terminus of *CaMK1* or *CaMK2* was transformed into hot pepper protoplasts by polyethylene glycol (PEG)-mediated transformation with *Tsi1-RFP* construct as nucleus-localization marker. GFP signals of GFP-CaMK1 and GFP-CaMK2 protein were merged with red signals of Tsi1-RFP but some GFP signals were also detected in the cytosolic region (Fig. 5a, panels ii and iii). These results demonstrate that CaMK1, CaMK2, and CaWRKYa show nucleus localization, suggesting that they could interact *in planta*.

To confirm the association between CaWRKYa and hot pepper MAPKs, CaMK1 and CaMK2 *in vivo*, we utilized the bimolecular fluorescence complementation (BiFC) assay in hot pepper protoplasts with modified BiFC constructs^37^. N-and C-terminal half fragments of yellow fluorescent protein (YFP) were fused to CaMK1, CaMK2, and CaWRKYa and YFP^N^-fused CaMK1 and CaMK2 were separately expressed in the hot pepper protoplasts along with CaWRKYa-YFP^C^. When the combination of constructs *YFP^N^-CaMK1* and *CaWRKYa-YFP^C^* were transiently coexpressed in hot pepper protoplasts by PEG-mediated transformation, reconstituted YFP fluorescence signal was detected in the nucleus by fluorescence microscopy analysis (Fig. 5b, panel i). The reconstituted YFP fluorescence signal was also detected in the nucleus when the combination of constructs *CaMK2-YFP^N^* and *CaWRKYa-YFP^C^* were coexpressed in hot pepper protoplasts (Fig. 5b, panel ii). The BiFC constructs of the basic leucine zipper 63 (bZIP63) transcription factor form homodimer in the nucleus. Constructed bZIP63-YFP^N^ and bZIP63-YFP^C^ were used as a positive control for BiFC assay (Fig. 5b, panel iii)^37^. To confirm reconstituted YFP fluorescence signals in the nucleus, co-immunoprecipitation (co-IP) assays were performed in hot pepper protoplasts with CaWRKYa and CaMK1/CaMK2. As in Fig. 5c, CaWRKYa co-IPs with both CaMK1 and CaMK2 *in vivo*. These results suggest that CaWRKYa could associate with CaMK1 and CaMK2 in the nucleus.

### CaMK1 and CaMK2 phosphorylated the SP clusters of CaWRKYa

**Figure 6:**
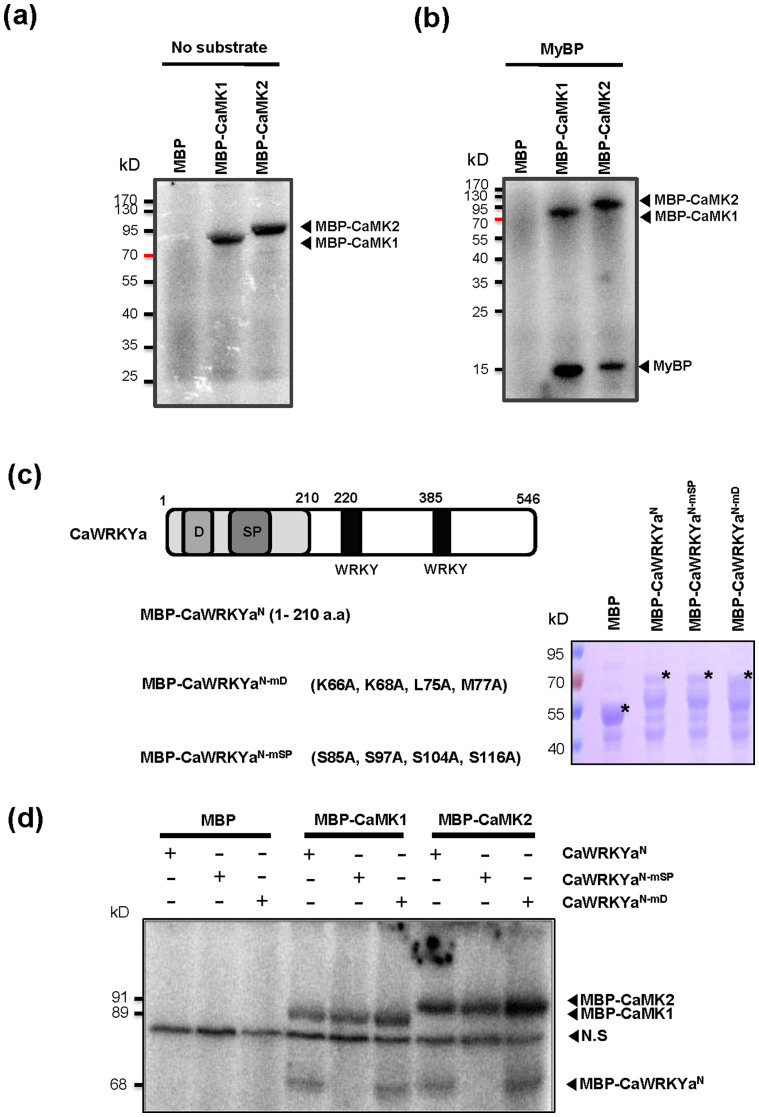
Analysis of CaWRKYa as a substrate of CaMK1 and CaMK2. (a) MBP-CaMK1 and -CaMK2 had an autophosphorylation activity. *In vitro* kinase assay was performed with MBP-CaMK1 (1 μg) and -CaMK2 (1 μg) incubated with no substrate, and MBP protein (1 μg) was used as a negative control. Proteins were incubated in the reaction buffer containing [γ-^33^P]-ATP for 1 h at RT, respectively. After heating for 5 min, the samples were run in SDS-PAGE gel. The gels were dried in gel dryer for 1 h and phosphorylation signals were detected by BAS reader. Arrows indicate phosphorylated CaMK1 and CaMK2. (b) MyBP protein (1 μg) was phosphorylated by MBP-CaMK1 and -CaMK2 *in vitro* kinase assay. Arrows indicate phosphorylated CaMK1, CaMK2 and MyBP. (c) A schematic illustration of *CaWRKYa* mutant constructs and confirmation of the purified proteins in SDS-PAGE gel. MBP-CaWRKYa^N^, MBP-CaWRKYa^N-mSP^ (S85A, S97A, S104A, S116A), and MBP-CaWRKYa^N-mD^ (K66A, K68A, L75A, M77A) proteins were made by mutagenesis. Purified proteins were checked via 15% SDS PAGE gel. Asterisk indicates specific band. (d) MBP-CaMK1 (1 μg) or -CaMK2 (1 μg) were incubated with CaWRKYa^N^ (1 μg), CaWRKYa^N-mSP^ (1 μg), and CaWRKYa^N-mD^ (1 μg) proteins and then *in vitro* kinase assay was performed. MBP was used as a negative control. N.S indicates non-specific bands.

To test whether CaMK1 and CaMK2 are catalytically active *in vitro*, the MBP-CaMK1 and MBP-CaMK2 fusion proteins were purified from *E. coli* (Supplementary Fig. S5a). Next the proteins were subjected to an *in vitro* auto-phosphorylation activity assay. Both MBP fusion recombinant CaMK1 and CaMK2 proteins exhibited auto-phosphorylation activity (Fig. 6a). To test if CaMK1 and CaMK2 are able to trans-phosphorylate the target protein the myelin basic protein (MyBP), which is a commonly used model protein substrate of MAPKs, was used as a substrate. MyBP was phosphorylated by CaMK1 and CaMK2 in the presence of [γ-^33^P]-ATP (Fig. 6b). Thus, recombinant CaMK1 and CaMK2 could be verified as catalytically active protein kinases that can phosphorylate MyBP as a substrate. Former results clearly demonstrated that CaMK1 and CaMK2 interacted with CaWRKYa *in vivo* (Fig. 5b and c). To assess the role of interaction between hot pepper MAPKs (CaMK1 and CaMK2) and CaWRKYa, we focused on the possibility of CaWRKYa as a substrate of CaMK1 and CaMK2. We expressed CaWRKYa^N^ fused with MBP protein to test the possibility that CaWRKYa acts as a substrate of CaMK1 and CaMK2 protein kinases (Fig. 6c). When affinity-purified MBP-CaWRKYa^N^ protein was incubated with each of CaMK1 and CaMK2 in the presence of [γ-^33^P]-ATP, both protein kinases could phosphorylate CaWRKYa^N^ *in vitro* (Fig. 6d), indicating that CaWRKYa is indeed a substrate of CaMK1 and CaMK2.

Some group I WRKY proteins contain the conserved D domain and SP clusters at the N-terminal region^15^ and CaWRKYa also has these domains (Fig. 1a). The D-domain is known to be important for the interaction of a substrate with MAPKs and phosphorylation of SP clusters^38^,^39^,^40^. To investigate the phosphorylation site of CaWRKYa, we made mutant protein of MBP-CaWRKYa N-terminal region, which was mutated either at the D-domain (mD) or SP clusters (mSP) (Fig. 6c). Interaction with MAP kinase could be achieved through the putative docking domain of CaWRKYa located at the N-terminus. Thus, if we mutate the corresponding region, we could expect significant decrease of enzymatic activity of both MAP kinases. However, WT CaWRKYa^N^ or CaWRKYa^N-mD^ protein were phosphorylated by recombinant CaMK1 and CaMK2 (Fig. 6d). These findings suggested that CaMK1 and CaMK2 might use non-identified docking motif in CaWRKYa. To further investigate the role of SP clusters for phosphorylation, a set of SP cluster mutants were constructed and tested for phosphorylation. MBP-fused recombinant CaWRKYa^N-mSP^ protein, which has four mutations in the SP cluster region (Fig. 6c), did not show phosphorylation signal by CaMK1 and CaMK2 (Fig. 6d). Recombinant MBP fused D-domain mutant CaWRKYa^N-mD ^was not affected in phosphorylation by CaMK1 and CaMK2 (Fig. 6d). These data indicated that the D-domain of CaWRKYa is not required for phosphorylation of CaMK1 and CaMK2 although interaction site is not clear in our experiments. However, multiple SP clusters of CaWRKYa have an important role in phosphorylation of CaWRKYa by CaMK1 and CaMK2.

A previous report suggested that phosphorylation of WRKY proteins affected DNA binding affinity of WRKY proteins to the *W*-box^15^. To test whether DNA binding affinity of CaWRKYa is enhanced by CaMK1 and CaMK2, we performed EMSA using purified GST fused with full length recombinant CaWRKYa protein with or without MBP-CaMK1 and -CaMK2 (Supplementary Fig. S5a). CaWRKYa incubated with CaMK1 or CaMK2 show slightly enhanced binding affinity to the *W*-box *in vitro* (Supplementary Fig. S5b). On the contrary, CaWRKYa with higher amount of CaMK1 was inhibited in DNA binding activity but CaMK2 still positively affects DNA binding affinity (Supplementary Fig. S5b). This result indicated that CaMK1 and CaMK2 function might be related to the regulation of stability and/or DNA binding affinity of CaWRKYa.

## Discussion

In the tobacco and TMV interaction model system, MEK1, NTF6, WRKY, and MYB are involved in *N*-mediated resistance to TMV^41^. This suggests that TMV resistance response is regulated by diverse regulators and especially that transcription factor-MAPK cascades might be a major regulation pathway of *R*-mediated resistance via posttranslational step. We have studied the hot pepper-TMV-P_0_ interaction model system and two WRKY proteins that were previously reported as being involved in *L*-mediated resistance to TMV-P_0_ via regulating *PR* genes expression^23^,^24^. In this study, we characterized CaWRKYa as a positive regulator in *L*-mediated resistance to TMV-P_0_ by regulating *PR* genes and SA/JA biogenesis genes. Furthermore, CaWRKYa was identified as a substrate of CaMK1 and CaMK2 in hot pepper. However, it is still largely unknown how other WRKY proteins are regulated by MAPKs or other regulators in hot pepper.

WRKY transcription factors are localized to the nucleus and then bind to *W*-boxes of target gene promoters to regulate gene expression upon stress conditions. However, WRKYs do not work alone in recognizing the signals from the stimulus. *R*-mediated signal transduction or MAPK signal cascade could be necessary for relaying the defense signal transduction to WRKYs or other transcription factors^42^. However, some WRKYs could directly regulate *R*-mediated signal transduction. The *slh1*mutant was isolated as a single amino acid insertion mutant in the WRKY domain of RRS1-R, and *slh1* showed enhanced disease resistance to pathogen infection^20^. Thus, this WRKY protein has evolved the capacity to play a dual role in defense signal recognition and regulation for plant immunity.

LeMPK3 can be activated in Cf-4/Avr4 interaction in tomato and is involved in HR upon pathogen infection^43^. WIPK is activated in *N* gene-mediated cell death in tobacco upon TMV infection^3^. CaMK2 also belongs to group A and shows high identity to LeMPK2 (95%), NTF4 (94%), and NtSIPK (88%) (Supplementary Fig. S2). LeMPK2 is required for prosystemin-mediated resistance to *Manduca sexta*^44^. NTF4 is known to be involved in pathogen-induced HR cell death in tobacco^45^. Furthermore, WIPK and SIPK also are activated by TMV and positively regulate *N* gene-mediated TMV resistance in tobacco^46^. Interestingly, NbWRKY8 was identified as a substrate of WIPK, SIPK, and NTF4. The D-domain of NbWRKY8 is known to be important for the interaction of these MAPKs^15^. Some group I WRKYs contain D-domain and SP clusters at the N-terminal region^17^. AtWRKY33 is also classified as a group I WRKY which contains D-domain and SP clusters. Similarly, AtWRKY33 is a substrate of MPK3/MPK6. However, phosphorylation of AtWRKY33 does not affect DNA binding affinity of AtWRKY33 to the *W*-box^16^,^47^. CaWRKYa is slightly affected in DNA binding affinity by CaMK1 and CaMK2 (Supplementary Fig. S5b). Furthermore, regulation of DNA binding affinity might be dependent on MAPK protein level because CaMK1 and CaMK2 differently regulated CaWRKYa DNA binding activity at higher protein level (Supplementary Fig. S5b). These results imply that MAPKs might regulate stability and/or transcriptional activation ability of WRKY proteins via phosphorylation and physical association.

CaWRKYa also contains D-domain and five SP clusters at the N-terminal region (Fig. 1a). We provided evidence that CaWRKYa is substrate of CaMK1 and CaMK2 (Fig. 6d). The SP cluster(s) of CaWRKYa is required for phosphorylation by CaMK1 and CaMK2 although the D-domain of CaWRKYa did not play an important role for phosphorylation by both CaMK1 and CaMK2 (Fig. 6d). Thus, WRKY-MAPK interactions seem to have been conserved in posttranslational regulation system but each specific interaction might need a platform of specific interaction motif of WRKY protein and it is still not clear. For instance, SIPK also phosphorylated NtWRKY1, which is classified as a group I WRKY, and transactivation activity of NtWRKY1 was enhanced although NtWRKY1 does not have the D domain^48^. *Arabidopsis* mitogen activated protein kinase kinase kinase 1 (MEKK1) can also phosphorylate AtWRKY53 which is classified as group IIIb *in vitro*^49^,^50^. Phosphorylated AtWRKY53 showed increased DNA-binding activity *in vitro* and *in vivo*^50^. AtWRKY53 contained four SP clusters at the N-terminal region but did not have a D-domain. Recently, CaWRKY58 was characterized as a negative regulator and classified as a group I WRKY. CaWRKY58 contained five conserved SP clusters but did not have D-domain^51^. CaWRKY2, another group I member, was isolated as a pathogen-inducible transcription factor and contained D-domain and five SP clusters although functional study of CaWRKY2 was not carried out^52^. These group I WRKYs should be further investigated in respect to WRKY-MAPK regulation system in hot pepper. So far, these results provide evidence that MAPKs could interact with D-domain of WRKYs but other unknown motifs of WRKYs might be also important for interaction of MAPKs. The mechanism of the regulation of WRKY proteins, especially group I members, via MAPKs cascade still leaves many questions and will be a subject of further investigations.

## Experimental procedures

### Plant materials and growth conditions

Pepper (*Capsicum annuum* L.) cultivar Bugang plants were grown on soil in a growth room at 25°C with 16 h light and 8 h dark photoperiod^23^.

### Virus-induced gene silencing (VIGS)

The partial 3' UTR region of *CaWRKYa* was cloned into the pTRV2 vector containing a part of the *Tobacco rattle virus* (TRV) genome using the primers; 5'-TATATGAATCAAATGCAGCCCACGAACA-3' and 5'-GCCAGGCTCAAAGACCAATAAAATAATG-3'. VIGS was performed with pTRV1 and pTRV2 or its derivatives in *Agrobacterium* strain GV3101^23^,^53^.

### Gene expression analysis upon the plant hormones treatments and TMV inoculation

TMV-P_0_ (avirulent) and PMMoV-P_1,2,3_ (virulent) strains were prepared with virus-containing sap in virus-inoculation buffer (25 mM phosphate buffer, pH 7.0) and inoculated on the 6-week-old hot pepper plant leaves by rubbing with carborundum (Hayashi Chemical). Samples were prepared at the indicated time points. Control plants were also treated with virus-inoculation buffer containing the carborundum only. For SA, MeJA, and ET treatments, 3-week-old plants were sprayed with solutions of 1 mM SA, 100 μM MeJA, and 1 mM ET, respectively. Control plants were sprayed with water containing 0.01% ethanol. Three independent samples were harvested and total RNA was prepared using Qiagen RNA kit. Quantitative RT-PCR was performed with SYBR Green (Kapa Biosystems) in the LightCycler 480 Real-Time PCR System (Roche Applied Science) according to the manufacturer's instructions. The primers used for real-time PCR reactions are listed in Table S1.

### Trypan blue staining

HR cell death was detected with trypan blue staining^23^. TMV-P_0_-inoculated leaves of TRV2 and TRV2-*CaWRKYa* plant were stained with lactophenol-trypan blue solution (10 ml lactic acid, 10 ml glycerol, 10 ml acidic-phenol, 0.02 g trypan blue, and 10 ml water) and chloral hydrate solution (Fluka chemical) was used as a destaining solution.

### Subcellular localization determination of CaWRKYa, CaMK1, and CaMK2

The *CaWRKYa*, *CaMK1*, and *CaMK2* coding regions were fused to the *green fluorescence protein* (*GFP*)-coding region at the N-terminal region in 326 GFP vector. The DNA of *GFP-CaWRKYa*, *GFP-CaMK1*, and *GFP-CaMK2* constructs was introduced into the hot pepper protoplasts by modified polyethylene glycol (PEG)-mediated transformation^54^. GFP signal was excited at 488 nm laser and was collected using 495-510 nm bandwidths using an Axioplan 2 imaging fluorescence microscope (Carl Zeiss).

### Bimolecular fluorescence complementation (BiFC) and co-immunoprecipitation (co-IP) assay

BiFC assays were performed as previously described^37^. Briefly, *CaMK1* clone was inserted into the pUC18-backboned vector which contains partial YFP-N fragment (1-155 amino acid) tagged with c-Myc at the N-terminus using the primers; 5'-GGATCCATGGTTGATGCAAATATGGGTGCGGCT-3' and 5'-GGATCCTTA AGCATATTCAGGATTCAGTACCAAGG-3'. The *CaMK2* clone was inserted into the pUC18-backboned vector which contains partial YFP-N fragment (1-155 amino acid) tagged with c-Myc at the C-terminus using the primers; 5'-GGATCCATGGATGGTCCAGCTCAGCAAACGG-3' and 5'-GGATCCATGTGCTGGTATTCGGGATTAAA-3'. The *CaWRKYa* clone was inserted into the pUC18-backboned vector which contains partial YFP-C fragment (155-239 amino acid) tagged with HA at the C-terminus using the primers; 5'-GGATCCATGGCTTCTTCAGGTGGAAATACG-3' and 5'-GGATCCGTTAAGGAAAGAGCTGAAGAATAAATC-3'. Combination of *CaWRKYa-YFP^C^* and YFP^N^-CaMK1 or *CaMK2-YFP^N^* constructs were transformed into the hot pepper protoplasts by PEG-mediated transformation. Reconstructed yellow fluorescent protein (YFP) signals was excited at 514 nm laser and was collected using 560–640 nm bandwidths using an Axioplan 2 imaging fluorescence microscope.

To confirm these BiFC interactions, co-IP was performed with the hot pepper protoplasts co-expressing *CaWRKYa-YFP^C^* and *YFP^N^-CaMK1* or *CaMK2-YFP^N^* constructs. Briefly, after PEG-mediated transformations into protoplasts from the hot pepper plant, the cells were harvested by quick spin. The cells were suspended in extraction buffer (150 mM NaCl, 50 mM Tris-Cl pH 7.5, 5 mM EDTA, 1% Triton X-100, 1 mM DTT, and proteinase inhibitor cocktail) and then total proteins were extracted by sonication. After centrifugation, the supernatants were mixed with HA-agarose beads and incubated at 4°C for 2 h. The samples were run in SDS-PAGE gel and then western blot was performed with ECL kit (Amersham).

### *In vitro* phosphorylation assay

The N-terminal CaWRKYa fragment (CaWRKYa^N^) protein and its mutant constructs were fused with maltose-binding protein (MBP) using pMAL vector. *CaWRKYa^N^* was amplified by PCR with primers; 5'-GGATCCATGGCTTCTTCAGGTGGAAATACG-3' and 5'-GGATCCTTAAACATATTGAGATGGTTGACGGTA-3'. To make WRKYa D-domain mutant (WRKYa^N-mD^; K66A, K68A, L75A, M77A) and SP cluster mutant (WRKYa^N-mSP^; S85A, S97A, S104A, S116A), we used PCR with primers; CaWRKYa^N-mD^-5'-GATGAGGTTCC AGCGTTCGCGTCTTTTCCACCTTCTTCTGCGCCTGCGATCTCTTCTTCA-3' and CaWRKYa^N-mD^-5'-TGAAGAAGAGATCGCAGGCGCAGAAGAAGGTGGAAAAGACGCGAACGCTGGAACCTCATC-3', CaWRKYa-S85AS97A-5'-TCATCACCAGCTGCTCCTTCTTCTTATCTTGCTTTTCCTCATTCTTTAGCTCCATCGGTTCTT-3' and CaWRKYa-S85AS97A-5'-AAGAACCGATGGAGCTAAAGAAT GAGGAAAAGCAAGATAAGAAGAAGGAGCAGCTGGTGATGA-3', CaWRKYa-S104S116-5'-GTTCTTTTGGACGCACCAGTTTTGTTTAACAATTCCAATACTCTTCCAGCACCAACAACAGGG-3' and CaWRKYa-S104S116-5'-CCCTGTTGTTGGTGCTGGAAGAGTATTGGAATTGTTAAACAAAACTGGTGCGTCCAAAAGAAC-3'.

For *in vitro* phosphorylation assay, MBP fused CaWRKYaN, CaMK1, and CaMK2 were expressed in *E*. *coli* and recombinant proteins were purified. MBP-CaMK1 (1 μg) or -CaMK2 (1 μg) were incubated with 1 μg of its substrates myelin basic protein (MyBP) or MBP-CaWRKYaN, -CaWRKYaN-mD, and -CaWRKYa-mSP proteins in reaction buffer (50 mM Tris-HCl pH7.5, 10 mM MgCl_2_, 2 mM MnCl_2_, 1 mM DTT, 50 μM ATP, [γ-^33^P]-ATP) for 1 h at RT. The reaction was stopped by adding the sample buffer. After heating for 5 min, the samples were run in SDS-PAGE gel and then stained and destained shortly. The gels were dried in gel dryer for 1 h and the signal was detected by BAS reader.

### Electrophoretic mobility shift assay (EMSA)

GST-fused full length CaWRKYa recombinant protein was purified according to the GST gene fusion system protocol provided by Amersham. EMSA was performed as described^55^. Briefly, double-stranded synthetic *W*-box and mutant *W*-box oligonucleotides were labeled with [γ-^33^P]-ATP using T4 polynucleotide kinase (TaKaRa). The labeled probes were incubated with purified GST-CaWRKYa protein (5 μg) or GST protein (5 μg) at 4°C for 30 min. DNA and protein complexes were resolved on a 5% polyacrylamide gel in 0.5 X TBE buffer. The gels were dried and the signals were detected by BAS reader.

### GUS promoter activity assay

*CaWRKYa*-*GFP* construct is used as an effector protein and *GFP* construct is used as a negative control. *CaPR10* *promoter*-*GUS* is used as a reporter^12^. Each effector and reporter was co-transformed into tobacco leaves by *Agrobacterium*-mediated infiltration. The leaves were harvested at three days after infiltration and extracted with GUS extraction buffer (50 mM NaHPO_4_ pH7.0, 10 mM 2-mercaptoethanol, 10 mM Na_2_EDTA, 0.1% sodium lauryl sarcosine, 0.1% Trition X-100) for GUS activity analysis. The 4-methyl-umbelliferyl-glucuronide (4-MU) as a substrate was used and protein concentrations of the extracts were determined by BSA assay. GUS activity was measured using a Mithras LB940-luminolmeter (Berthold Technologies)^23^. The excitation wavelength was 365 nm and the emission wavelength 455 nm.

### Electrolyte leakage assay

The electrolyte leakage assay was performed as described previously^23^. Briefly, three leaf discs were taken from one leaf of each TMV-P_0_ inoculated plant. The conductivity is measured by portable conductivity meter (Thermo Orion). The error bars show the mean value of the standard deviation (SD) of the replicate samples (n = 3).

## Supplementary Material

Supplementary Information Additional information
