# Supplementary material for: Capsicum annuum transcription factor WRKYa positively regulates defense response upon TMV infection and is a substrate of CaMK1 and CaMK2
Source: Sci Rep. 2015 Jan 23;5:7981. doi: 10.1038/srep07981 (PMC5379037; doi:10.1038/srep07981)
Supplement: Supplementary Information — Additional information [file srep07981-s1.pdf]

Capsicum annuum transcription factor WRKYa positively regulates defense response upon TMV infection and is a substrate of CaMK1 and CaMK2

Sung Un Huh[1], Gil-Je Lee, Ji Hoon Jung, Yun-Sik Kim, Young Jin Kim & Kyung-Hee Paek*

College of Life Sciences and Biotechnology, Korea University, 1, 5-ga, Anam-dong, Sungbuk-gu, Seoul 136-701, Republic of Korea

Corresponding author: Kyung-Hee Paek*

College of Life Sciences and Biotechnology, Korea University, 1, 5-ga, Anam-dong, Sungbuk-gu, Seoul 136-701, Republic of Korea

Tel: 82-2-3290-3440; Fax: 82-2-928-1274

E-mail: khpaek95@korea.ac.kr

[1]Current address: The Sainsbury Laboratory, Norwich Research Park, Norwich NR4 7UH, United Kingdom

Additional information

Competing financial interests: The authors declare no competing financial interests.

Figure legends (Figures S1-S5 and table S1)

Fig. S1 GST-CaWRKYa had a DNA binding affinity for *W*-box. (a) GST fused CaWRKYa full-length protein was purified through GST purification column and checked in 15% SDS-PAGE gel. (b) EMSA was performed with *W*-box and mutant *W* (m*W*)-box synthetic oligonucleotides. GST-CaWRKYa (5 μg) protein was incubated with [γ-^{32}P]-ATP labeled *W*-box or m*W*-box and complexes were electrophoresed in 5% native acrylamide gel. The non-radiolabeled *W*-box or m*W*-box oligonucleotides were used as competitors. (c) Transactivation activity analysis of *CaPR10 promoter-GUS* gene expression by CaWRKYa-GFP in tobacco plants. Leaf discs were harvested 72 h after agroinfiltration. GUS activity was measured using a Mithras LB940-luminolmeter (Berthold Technologies). The error bars show the mean value of the standard deviation (SD) of the replicate samples (n=3).

Fig. S2 Analysis of CaMK1 and CaMK2 amino acid with other MAPKs from various plants. (a) CaMK1 and CaMK2 amino acids were aligned with other species MAPKs by MegAlign software. Threonine and tyrosine motif (TEY) which is a phosphorylation-activation motif was conserved (red box). Accession numbers; OsMPK3 (AF216317), OsMPK6 (ABO69383), NTF4 (X83880), AtMPK6 (D21842), AtMPK3 (D21839), LeMPK2 (NP_001234355), LeMPK3 (AAP20421), StMAPK (BAE44363), AtMPK4 (D21840), SIPK (D21830), WIPK (D61337), CaMK1 (AF247135), CaMK2 (AAF81420), OsBIMK1 (AF332873), AtMPK5 (D21841), AtMPK1 (D14713), AtMPK2 (D14714), AtMPK7 (D21843), AtMPK9 (NM112686), BWMK1 (AF177392). (b) Phylogenetic analysis of CaMK1 and CaMK2 with other MAPKs by Clustal W method. CaMK1 and CaMK2 are classified as group A MAPK.

Fig. S3 Expression analysis of *CaMK1*, *CaMK2*, and *CaWRKYa* in plant defense-related hormone treatments. (a-d) Three-week-old hot pepper plants were sprayed with 1 mM SA, 100 μM MeJA, and 1 mM ET. Mock buffer containing 0.01% EtOH was also sprayed in the hot pepper plants. Samples were harvested at the indicated time points and total RNAs were extracted from three independent samples. qRT-PCR was performed with gene-specific primers (Table S1). *Ca18S* gene was used as an internal control. (Student's *t*-tests; *$P < 0.05$, **$P < 0.001$, ***$P < 0.0001$).

Fig. S4 Silencing effects of *CaMK*1 and *CaMK2* upon TMV-P_0 infection. (a) To ensure efficient VIGS, RT-PCR was performed with the silenced hot pepper plants upon TMV-P_0 inoculation. The gene-specific region of *CaMK1* and *CaMK2* targeted for silencing was used for TRV-based VIGS. An empty vector TRV2 was applied to plants as a negative control. (b) Cell death lesions in TRV2, TRV2-*CaMK1*, and -*CaMK2* silenced hot pepper plants upon TMV-P_0 infection at 3 dpi. Leaves were decolorized in ethanol to visualize HR cell death. Numbers of HR lesions were quantified by counting (Student's *t*-tests; **$P < 0.001$, ***$P < 0.0001$).

Fig. S5 Effect analysis of CaMK1 and CaMK2 for DNA binding affinity of CaWRKYa. (a) Recombinant GST-CaWRKYa, MBP-CaMK1, and MBP-CaMK2 proteins were purified and protein expression was confirmed by SDS-PAGE gel. Purified proteins are indicated by asterisks. (b) GST-CaWRKYa protein was mixed with MBP, MBP-CaMK1, and MBP-CaMK2 and then incubated with [γ-^{32}P]-ATP-radiolabeled *W*-box probe. In 5% native PAGE gel, ESMA was performed. The relative signal intensity was calculated by ImageJ (imagej.nih.gov/ij/).

Table S1. RT-PCR and qRT-PCR primer list

Supplementary Figure S1

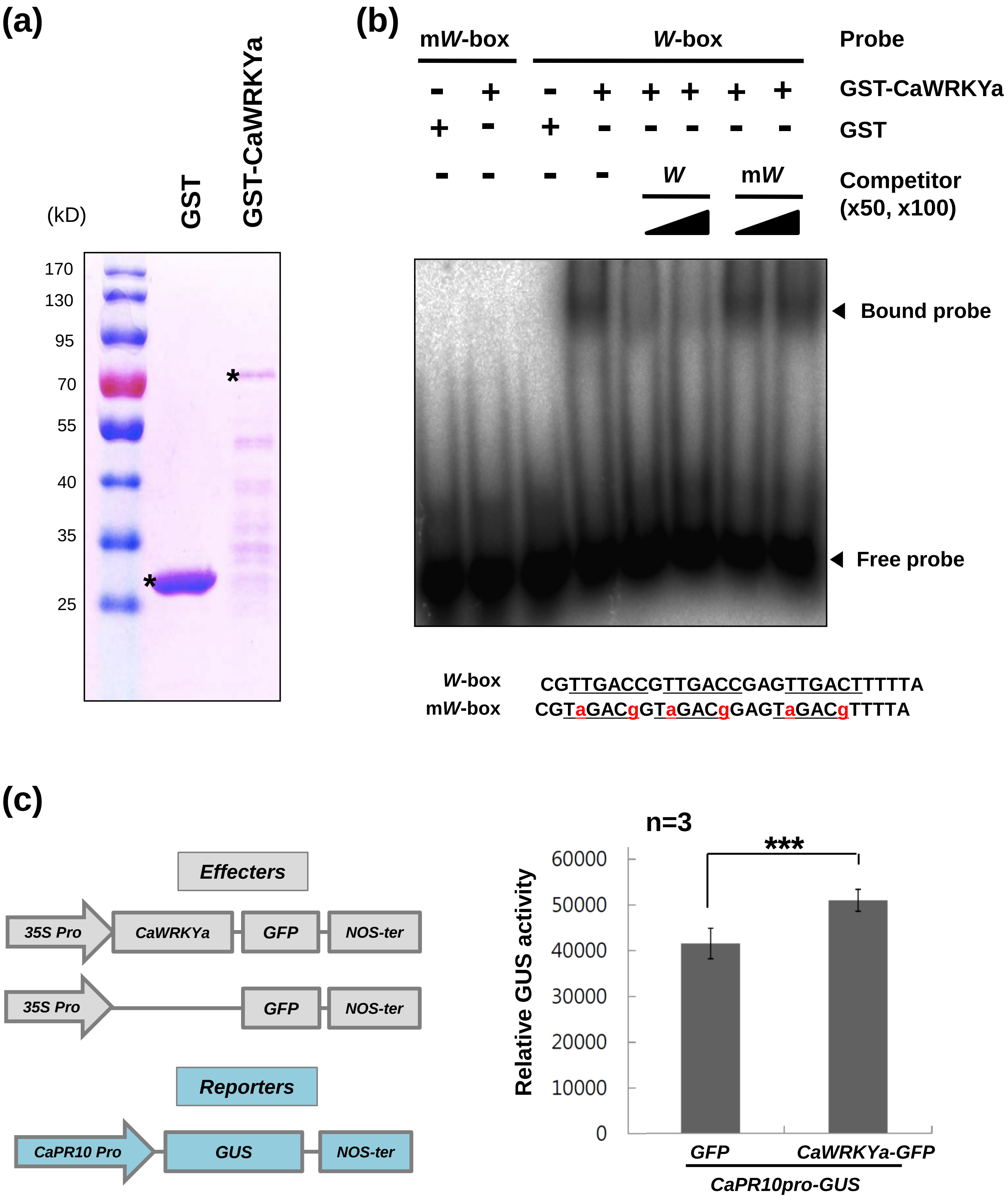

Supplementary Figure S2

(a)

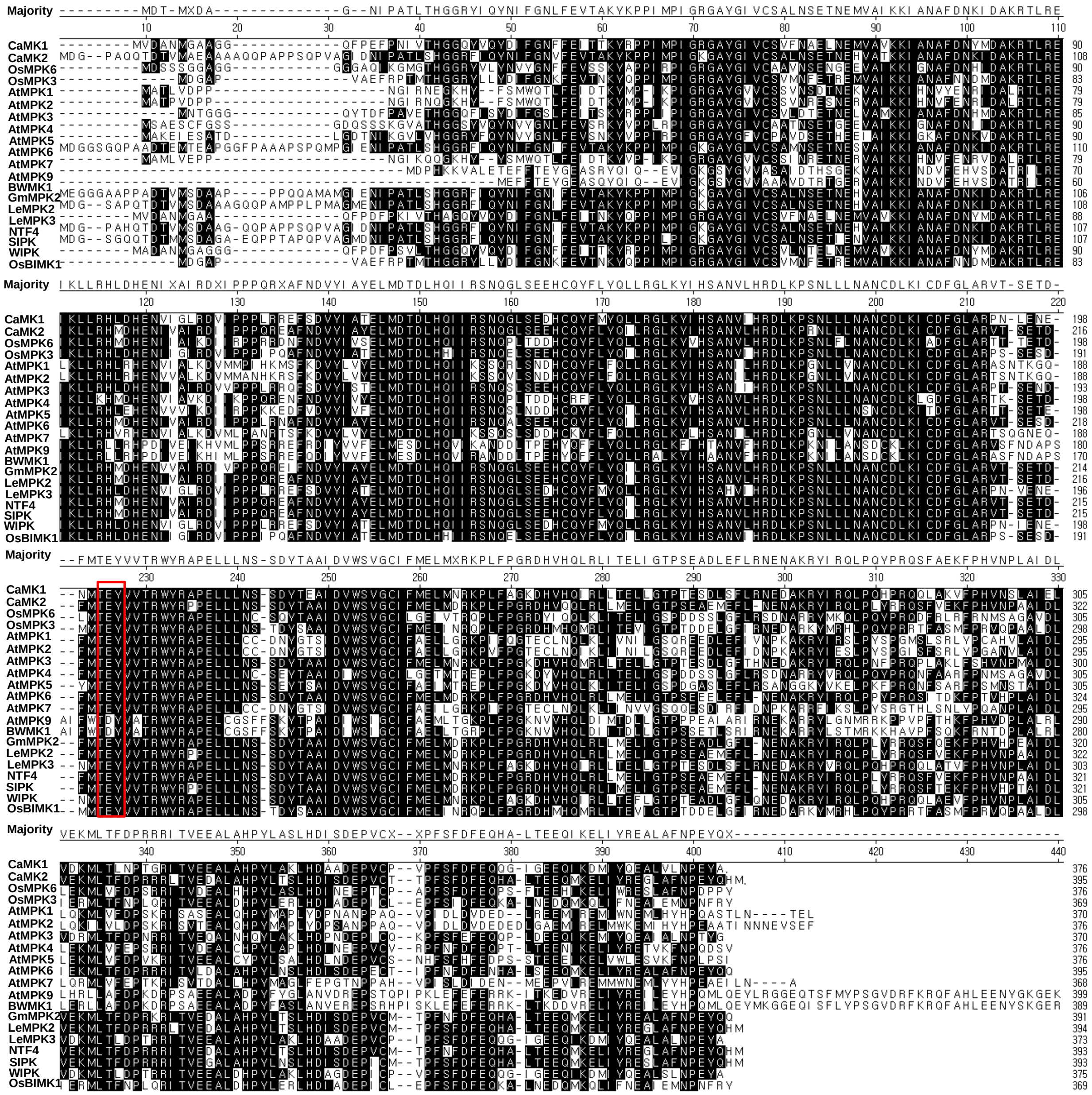

(b)

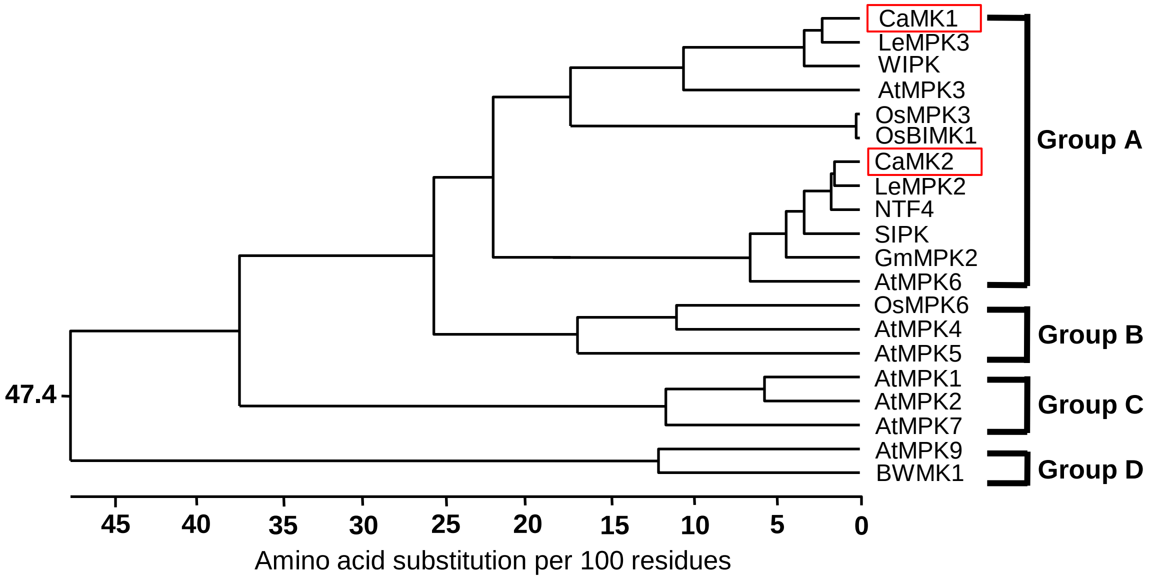

Supplementary Figure S3

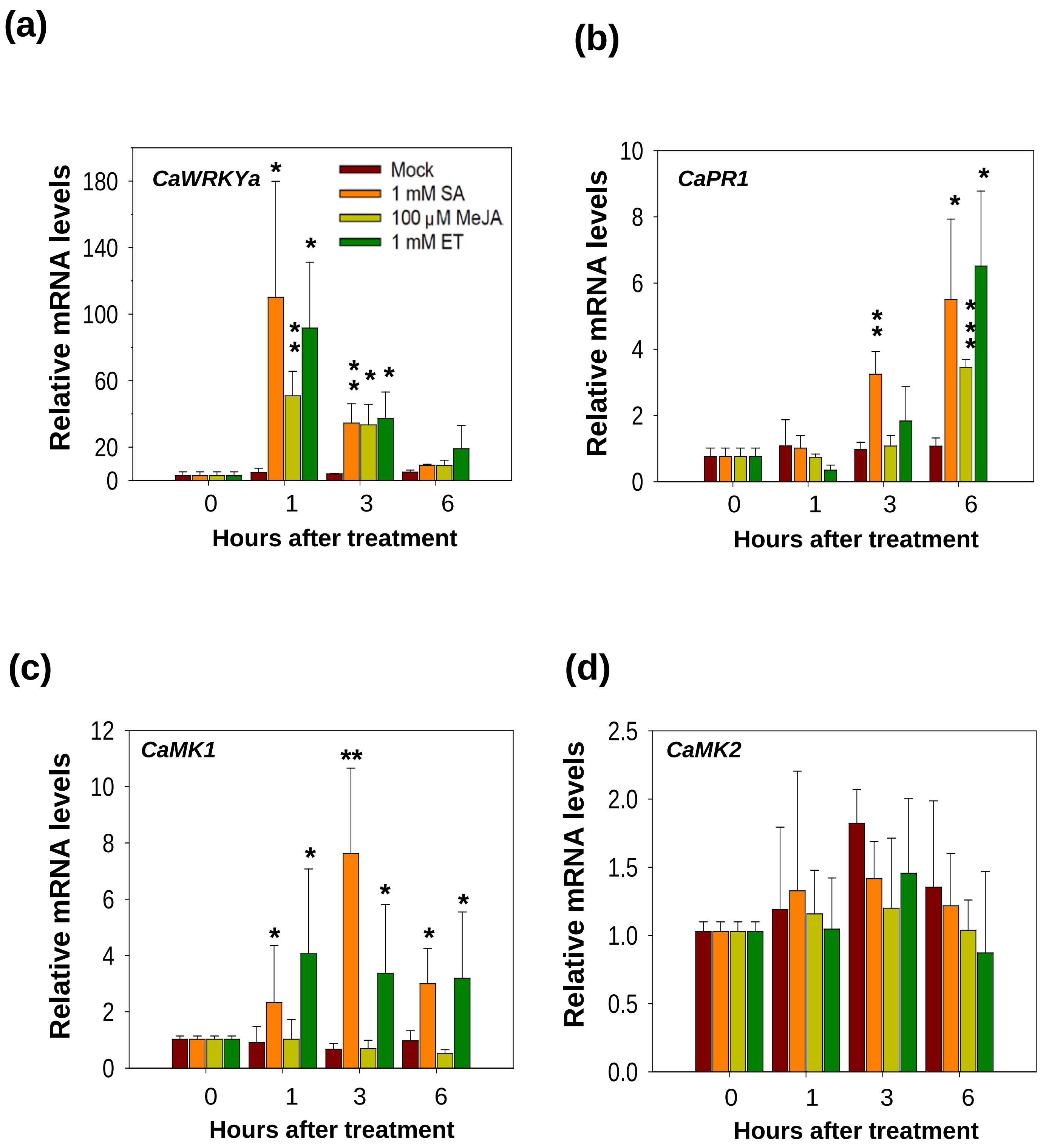

Supplementary Figure S4

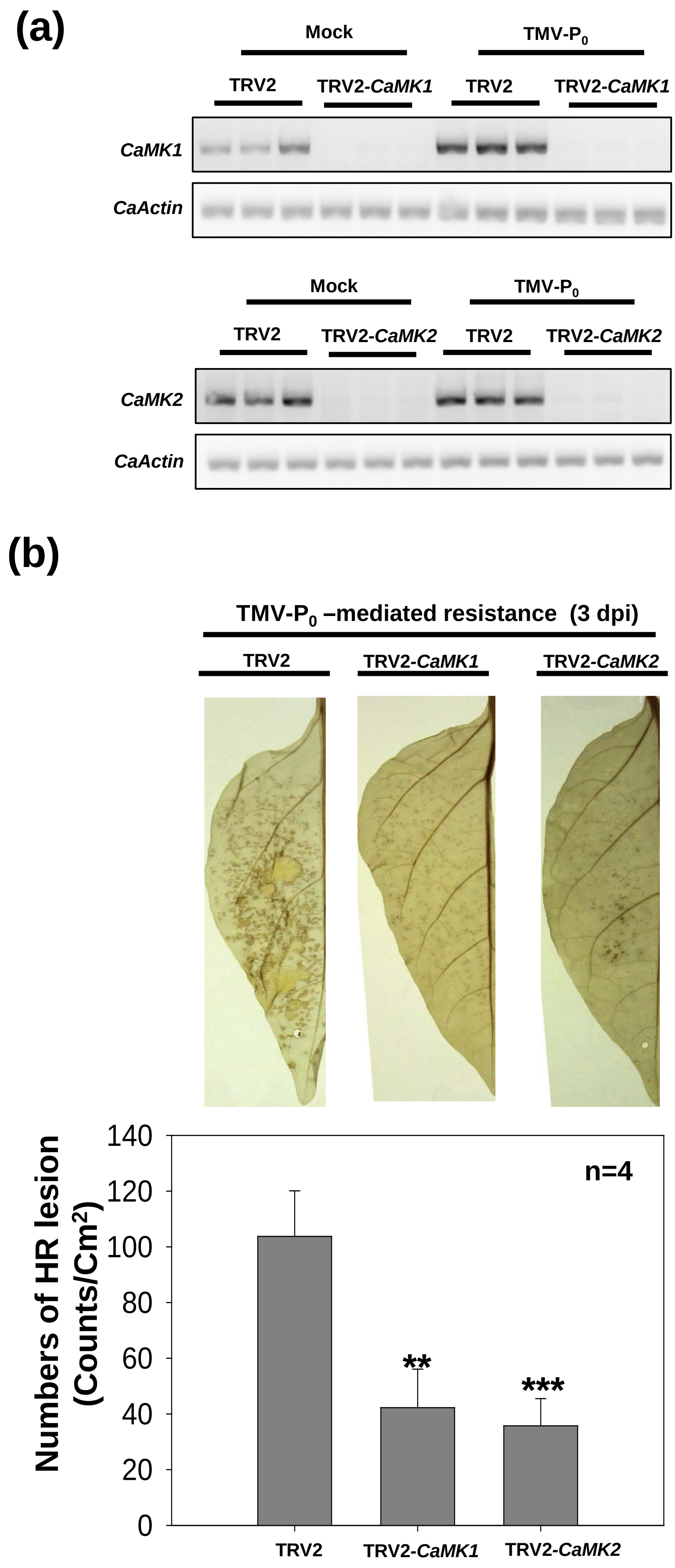

Supplementary Figure S5

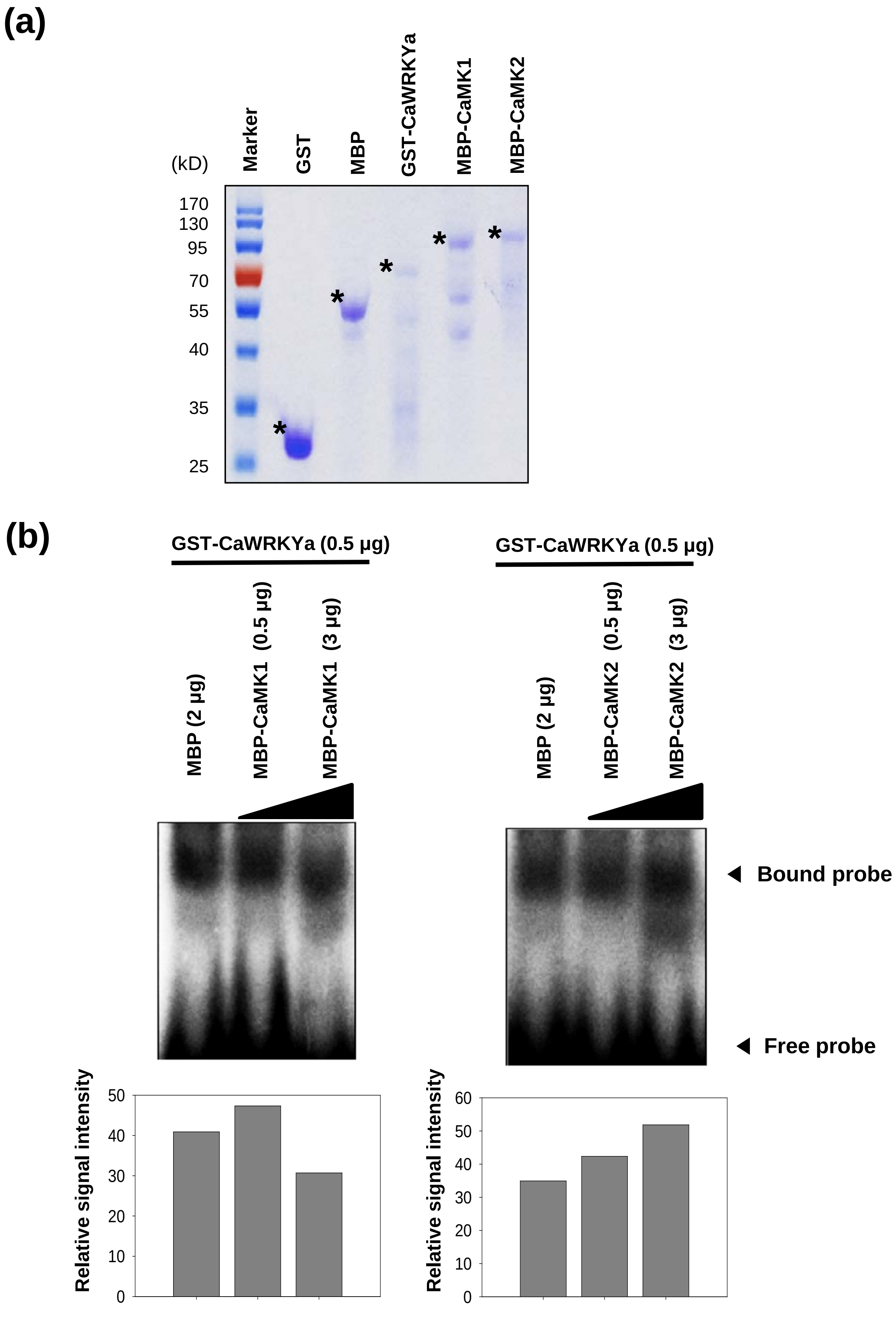

Supplementary Table S1 RT-PCR and qRT-PCR primer list

Gene name	Forward primer (5' to 3')	Reverse primer (5' to 3')
TMV-P_0-*CP*	TAGACCCGCTAGTCACAGCA	GAGGTCCAAACCAAACCAGA
CaActin	ATTGTCTTGAGTGGTGGTTCT	TTCCTCTCTGGTGGTGCTAC
Ca18S	AAACGGCTACCACATCCAAG	ACCCATCCCAAGGTTCAACT
CaPR1	GACATGGGACAATAGGCTAG	CAGTTGGAAGTTCCAATTTG
CaPR2	CTACTTAAGCTTTGCAAGACACCA	AGATCTCTTTCCTCATCGTCACTT
CaPR4	GAACACAAGCAACGGTGAGA	GGCACTTGTTTAGGCAGAGC
CaPR5	TTGCCAAAGTTGGACTACTGATTA	TCAACCAAATTGAACAAAAAGAGA
CaPR10	GGCAAATTTGAAGCTTCTGC	AAGGATTGGCGAGGAGGTAT
CaWRKYa	AATTACGAATTCAATTAACAAAGAT	ATGGAAGAGTATTGGAATTGTTA
CaMK1	CGGCTGGTGGTCAATTCCCTG	CATTAAGCAAAAGGTTGCTT
CaMK2	GGACACCGTGATGGCAGAG	TAGAGCGAATAATTTGATGGAGAT
CaNDR1	ACATTGTTAGAGTCCTCCATCACC	CCCTCGACATGAACCCTAAAA
CaICS1	AATGCTCCCTTTCCTCTTCACC	TCTATTGCCCTCTGGTATGTCC
CaLox2	ATCCTTATGCCGTATGTGAGGA	AGCAGCGTTGATTGAGATGTGT
